# Research on path planning of autonomous manganese nodule mining vehicle based on lifting mining system

**DOI:** 10.3389/frobt.2023.1224115

**Published:** 2023-07-27

**Authors:** Yingchun Xie, Chaoqun Liu, Xuguang Chen, Guijie Liu, Dingxin Leng, Wei Pan, Shuai Shao

**Affiliations:** College of Engineering, Ocean University of China, Qingdao, China

**Keywords:** autonomous manganese nodule mining vehicle, manganese nodule, path planning, sweeping coverage, obstacle avoidance algorithm

## Abstract

Deep-sea manganese nodules are abundant in the ocean, with high exploitation potential and commercial value, and have become mineral resources that coastal countries compete to develop. The pipeline-lifting mining system is the most promising deep-sea mining system at present. A deep-sea mining vehicle is the core equipment of this system. Mining quality and efficiency rely on mining vehicles to a great extent. According to the topographic and geomorphic environmental characteristics of deep-sea manganese nodules at the bottom of the ocean, a new deep-sea mining system based on an autonomous manganese nodule mining vehicle is proposed in this paper. According to the operating environment and functional requirements of the seabed, a new mining method is proposed, and the global traverse path planning research of the autonomous manganese nodule mining vehicle based on this mining method is carried out. The arc round-trip acquisition path planning method is put forward, and the simulation verification shows that the method effectively solves the problems of low efficiency of mining vehicle traversing acquisition and obstacle avoidance.

## 1 Introduction

Manganese nodules are widely distributed on the seabed surface at a depth of 2,000–6,000 m in the world’s major oceans. They mainly occur in deep-sea plains, platforms, trenches, and other areas. Two-dimensional sediments are formed within 10 cm of the top or shallow layer of deep-sea sediments, which exist in the form of potato-like stones. Manganese nodules are rich in more than 20 kinds of metallic elements and dozens of other non-metallic elements, with huge reserves, of which four kinds of metals, manganese, copper, nickel, and cobalt, are equivalent to hundreds or even thousands of times total land reserves (Meiyi, 2018).

In order to use the rich mineral resources of deep-sea manganese nodules for human production and development, several countries have experienced decades of efforts and proposed seabed towing mining systems, continuous chain bucket mining systems (Masuda et al., 1971), shuttle boat mining systems (Rajesh et al., 2011), and pipeline lifting mining systems (Sharma, 2017). After several offshore tests, the pipe-lifting mining system is currently considered to have the most practical application value and development potential (Wu et al., 2020). The system continuously collects manganese nodules by using deep-sea mining vehicles (or mining robots) to travel on the soft soil of the seabed. The pipeline connecting the surface mother ship and the deep-sea mining vehicles is the transportation channel to transport manganese nodules from the seabed to the surface. Among them, the deep-sea mining vehicle is the core equipment for collecting manganese nodules. Although the deep-sea mining vehicle can collect manganese nodules on the seabed at present, the global path planning research is not deep enough, and it is difficult to collect manganese nodules in a region with high efficiency, high quality, and a high collection rate. Therefore, in view of the problem of the high collection rate of deep-sea mining vehicles, it is also necessary to study reasonable mining methods.

Path planning is the primary guarantee for deep-sea mining vehicles to traverse the manganese nodule area on the seabed, and its planning accuracy directly affects the operation’s efficiency. Path planning can be divided into local path planning and global path planning. Local path planning, it is more focused on considering the current local environment information of the robot. The working environment of the robot is detected by the sensor to obtain the position and geometric properties of the obstacle so that the robot has good obstacle avoidance ability. This kind of planning needs to collect environmental data and realize the dynamic update of the environmental model in real time. However, it requires the robot system to have high-speed information processing ability and computing ability, and high robustness to environmental errors and noise. However, due to the lack of global environmental information, the planning results may not be optimal. For global path planning, global path planning is to planning a path for the robot in a known environment. The accuracy of path planning depends on the accuracy of environmental acquisition. Therefore, the real-time computing ability of the robot system is not required, and the obtained planning results are global and better. Considering the economic benefits and safety of mining, the path planning of deep-sea mining vehicles should meet the requirements of a high acquisition rate and effective obstacle avoidance. At present, global path planning has been widely used by robots in the fields of sea, land, and air. Some applications such as ground cleaning (Oh et al., 2001; Dang et al., 2013; Hsu et al., 2014), mine detection and clearance (Najjaran and Goldenberg, 2005; Hameed, 2016) weed removal, and automatic crop harvesting are global path planning applications on a two-dimensional plane (Ullrich et al., 2012; Galceran and Carreras, 2013; Prado et al., 2013). More complex applications are global path planning applications in three-dimensional space such as automatic painting (Atkar et al., 2005) or underwater inspection (Tao et al., 2008; Ergezer and Leblebicioğlu, 2014; Torres et al., 2016). Since most areas of manganese nodules are similar to plains and the slope is 0–3°, the mining area can be regarded as a two-dimensional plane, and the path planning of deep-sea mining vehicles can be simplified to be carried out on a two-dimensional plane. Chen et al. (2020) proposed optimal path planning for seabed mining vehicles based on the A* algorithm. They constructed 10,000 m^2^ simulated topographic maps of polymetallic mining areas and change the searchable nodes of the A* algorithm. The penalty coefficient is introduced to consider the uncertainty in the turning of the mining vehicle, and the feasible obstacle avoidance scheme in the mining area is obtained by taking the turning times, time consumption, path consumption, and path smoothness as the evaluation criteria (Chen et al., 2020). Xuan (2019) combined the improved A* algorithm with APF. Firstly, the global path was obtained through the A* algorithm, then the inflection point generated by the A* algorithm was taken as the starting point and the ending point, and the improved APF was used as the local planning, so that the generated path could be close to the optimal path (Xuan, 2019). Park et al. (2011) improved the traditional right-angle turning path planning method. In order to reduce the time and the number of turns consumed by the processes of deceleration, turning, and acceleration in the right-angle turning of the mining robot, a slightly angled arc turning was proposed, which saved time (Park et al., 2011). However, the arc of the turning is small, and the difference between the two sides of the robot’s tracks is large. The disturbance to the seabed’s rare soft soil is still large, which is easy to cause the robot to sink, reduce work efficiency and even lose workability (Pratama, 2015) studied the operation of underwater mining robots on the seabed of uneven terrain and proposed a method to minimize the overlap path and the number of turns. This method generates a coating cover path with short overlap, a few turns, and fast coating over time. However, using this path planning method, the mining efficiency is not high (Song and Arshad, 2016) proposed a pre-task coverage path planning method. Based on the investigation of the currently available coverage path, the inspection path was constructed. Although the comprehensive coating coverage path can be realized and the required operation time can be reduced, it still causes great disturbance on the seabed rare-soft soil when it is used in the collection of manganese nodules (Liang et al., 2021) used a method of mixing cuckoo search and a bat algorithm is used so that the underwater robot can reach a target point by means of the shortest path while avoiding obstacles (Li et al., 2022) proposed an improved A * algorithm, and compared it with the A* algorithm, the genetic algorithm (GA) and the simulated annealing algorithm (SA) in terms of the computation time, path length, and turning angle. The results reveal that the proposed algorithm can solve the robot path planning problem more efficiently and smoothly than the other algorithms mentioned above (Wang et al., 2022) proposed an improved A*-based algorithm. called the EBS-A* algorithm, which introduces expansion distance, bidirectional search, and smoothing into path planning. The results show that compared with the traditional A* algorithm, the proposed algorithm improves the path planning efficiency by 278% and reduces the number of critical nodes by 91.89% and the number of right-angle turns by 100%. Considering the existing full coverage path planning method of deep-sea manganese nodule mining robots, the problem of disturbance of rare soft soil in mining areas has not been solved. A new full coverage path planning method is proposed to solve this problem.

In this paper, a mining method and path planning method for autonomous manganese nodule mining vehicles are proposed based on the current recognized lifting mining system. A circular mining area with a connection center at the center is stipulated. The circular area is divided into six equal fan areas, and multiple mining vehicles are used to cooperate at the same time to achieve the purpose of high-efficiency mining work. Through the analysis of the abundance characteristics of manganese nodules in the mining area and the parameters of the fan-shaped mining area, the acquisition path in the recoverable area of manganese nodules is designed in advance, and the terrain slope and undulation of the mining area are extracted to prepare for the later algorithm. By analyzing the constraints of the mining vehicles and the principle of obstacle avoidance algorithm, path planning is simulated to verify the passing ability and obstacle avoidance effect of this path planning method. The collection path performance of the path planning method and the protective effect on seabed soil is evaluated by calculating the path collection repetition rate. Increasing the turning radius to reduce the track differential to reduce the damage to the seabed soil. The results show that the path coverage rate designed in this paper is large, and the repeated acquisition rate and missed acquisition rate are low. It can be seen that the path planning scheme proposed in this paper has high efficiency and is suitable for deep-sea manganese nodule mining.

## 2 Scheme design of new deep sea mining system

**FIGURE 1:**
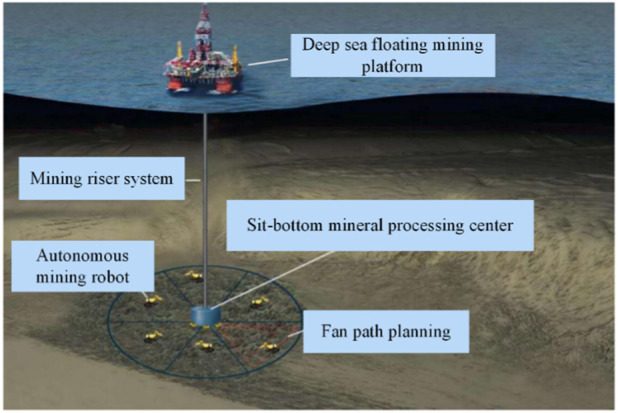
Concept map of the operation mode of deep-sea mining system.

Due to the flat seabed, manganese nodules have the largest mining potential and are easy to implement. According to the occurrence environment and state of manganese nodules, a new operation mode of a deep-sea mining system is proposed, as shown in Figure 1. As a mining tool, the autonomous mining robot (DSAMV, hereinafter referred to as the mining vehicle) automatically collects manganese nodules in the planned fan-shaped sub-region. After full storage, the manganese nodules are transported to the bottom-seated mineral connection processing center and unloaded. The energy is supplemented according to the required endurance. The empty vehicle returns to the designated mining area for further mining. Manganese nodules collected by the sitting-bottom mineral processing center are transported to the deep-sea floating mining platform by the mining riser system.

The mining vehicle works in a large circular mining area with a given radius based on the connection processing center. Several transponders are arranged in this large circular mining area, and acoustic transducers are installed on the mining vehicle so that it can be accurately positioned. The whole large circular mining area is divided into six 60° fan-shaped sub-regions, and six mining vehicles continuously collect manganese nodules in the designated fan-shaped sub-region according to the predetermined route without affecting each other.

Before the mining vehicle is mined, the regional topographic map of the whole large circular mining area is drawn first, and the topographic data of the mining area is uploaded to the acquisition center and shared with all the mining vehicles. According to these data and the real-time collected terrain and obstacles, the mining vehicle plans the whole covering path and traverses the entire mining area.

## 3 Mining vehicle path planning scheme

The most important part of the entire mining process of transporting manganese nodules from the seabed to the sea using the mining system scheme described above is the mining and transport efficiency of the mining vehicle. A reasonable and efficient mining path planning can save a lot of energy and maximize the exploitation of manganese nodules, reducing the waste of missing manganese nodules caused by low coverage or the waste of efficiency caused by high repetition rates during mining.

### 3.1 Abundance characteristics and parameter analysis of fan-shaped mining area

**FIGURE 2:**
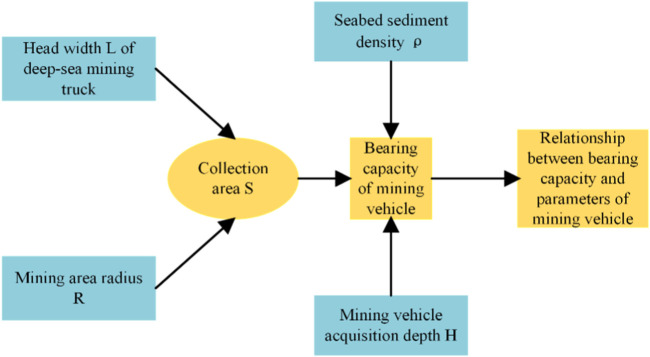
Indication diagram of circular mining area parameters.

To achieve quantitative mining in a fan-shaped sub-region, it is necessary to determine the relationship between the range parameters of the mining area and the bearing capacity of the mining vehicle, as shown in Figure 2.

According to the geological conditions of the contract area in the Pacific Ocean, the average abundance of nodules in the mining area is 10 kg/m^2^. The bearing capacity formula of a single mining vehicle can be expressed as:

$$G=\frac{\pi R^{2}}{6}\cdot \gamma \cdot \eta \cdot \mu$$ (1)

Where *G* is the bearing capacity of the mining vehicle and *R* is the radius of the circular mining area; where γ is an average abundance of manganese nodules in the mining area and η is the proportion of effective acquisition area in the fan-shaped sub-mining area; where μ is mining vehicle collection efficiency.

According to Eq. 1, as well as the sediment density and nodule abundance of the application mining area for ocean polymetallic nodules in China in Tables 1, 2, the corresponding relationship between the size of the circular mining area and the bearing capacity of mining vehicles can be determined.

**TABLE 1:** Sediment density of the mining area applying for ocean polymetallic nodules in China.

Sediment depth [m]		Moisture content [%]	Wet density [t·m^−3^]	Dry density [t·m^−3^]
5	East	81.8	1.20	3.1
	West	73.8	1.19	3.17
20	East	72.1	1.23	3.6
	West	68.3	1.24	3.7
60	East	64.9	1.25	3.8
	West	65.4	1.25	3.8
Mean range		70–82	1.20–1.25	3.1–3.8

**TABLE 2:** The abundance distribution of nodules in China’s ocean polymetallic nodules application mining area.

Abundance [kg/m^2^]		0–2.5	2.5–5.0	5.0–7.5	7.5–10.0	10.0–12.5	12.5–15.0	15.0–17.5	17.5–20.5	>20
Distribution frequency [%]	West	23.15	24.11	26.01	13.84	9.79	1.67	1.43		
	East	7.41	11.11	12.73	14.58	18.98	16.44	11.81	6.01	0.93

### 3.2 Design of acquisition path in the recoverable area of manganese nodules

The mining vehicles are located in a large circular area with the base-type mineral connection processing center as the origin. Six mining vehicles work independently in their respective 60° sector sub-region. Therefore, the simplified model is described as the mining vehicle in the 60° sector sub-region. Two path planning methods are proposed, radius round-trip acquisition path and arc round-trip acquisition path, and the two methods are deeply studied and analyzed.

#### 3.2.1 Radius round-trip acquisition path

**FIGURE 3:**
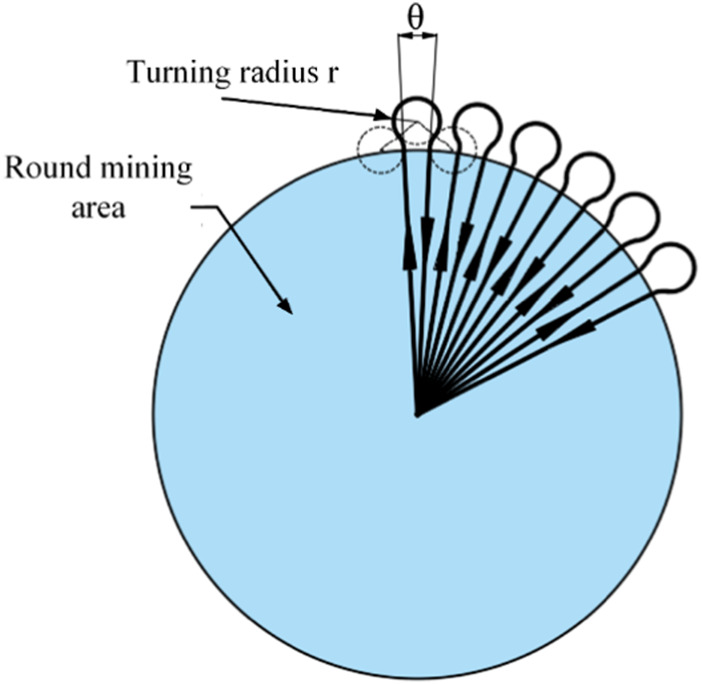
Diagram of radius round trip acquisition path.

In view of the acquisition efficiency of the mining vehicle, it is necessary to design the expected acquisition path in the recoverable area. Firstly, a radius round-trip acquisition path is proposed, as shown in Figure 3, which is the schematic diagram of the radius round-trip acquisition path. The mining vehicle starts from the connection center and mines along the radius direction. When it reaches the edge of the circular mining area, the large turning radius is used to adjust the speed. Then, it continues to mine in the radius of the mining area and returns to the connection center to unload the minerals. This path requires the mining vehicle to turn around the outside of the circular mining area, which can realize the full cover of the mining area. The round-trip acquisition of the mining vehicle can ensure the continuity of the mineral acquisition operation and the constant weight of the mineral.

**FIGURE 4:**
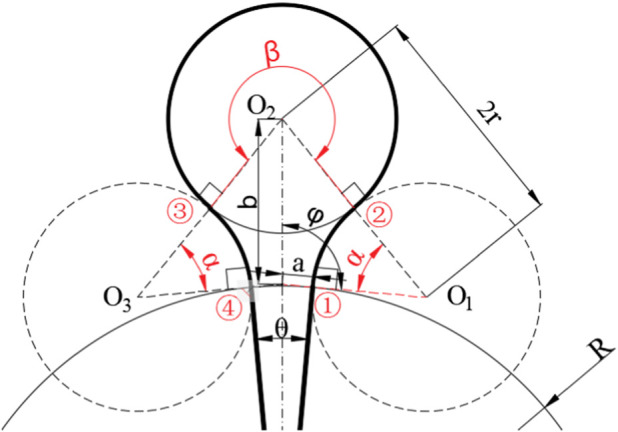
Large turning radius turning head schematic.

Figure 4 is the analysis chart of the large turning radius of the mining area boundary. The boundary turning has four times speed regulation. According to the speed ratio of the two sides of the track and the speed value of the low-speed side, the large turning radius of the mining vehicle can be calculated. Combined with the angle between the round and round paths and the radius R of the circular mining area, the arc angle of several turning can be calculated.

The radius round-trip acquisition path is calculated and analyzed: assuming the angle of a fan-shaped sub-region, the radius of the mining area is R, and the acquisition width of the mining vehicle is L.

Single acquisition area:

$$S_{c}=\frac{L}{2}\sqrt{R^{2}-\frac{L^{2}}{4}}+R^{2}\arcsin\frac{L}{R^{2}}\sqrt{R^{2}-\frac{L^{2}}{4}}$$ (2)

The overlapping area of adjacent paths:

$$S_{R}=\frac{L}{2}\sqrt{R^{2}-\frac{L^{2}}{4}}$$ (3)

The angle of the round trip path:

$$\theta =\arcsin\frac{L}{R^{2}}\sqrt{R^{2}-\frac{L^{2}}{4}}$$ (4)

Where θ is the central angle corresponding to the width of the mining head as the chord length.

**FIGURE 5:**
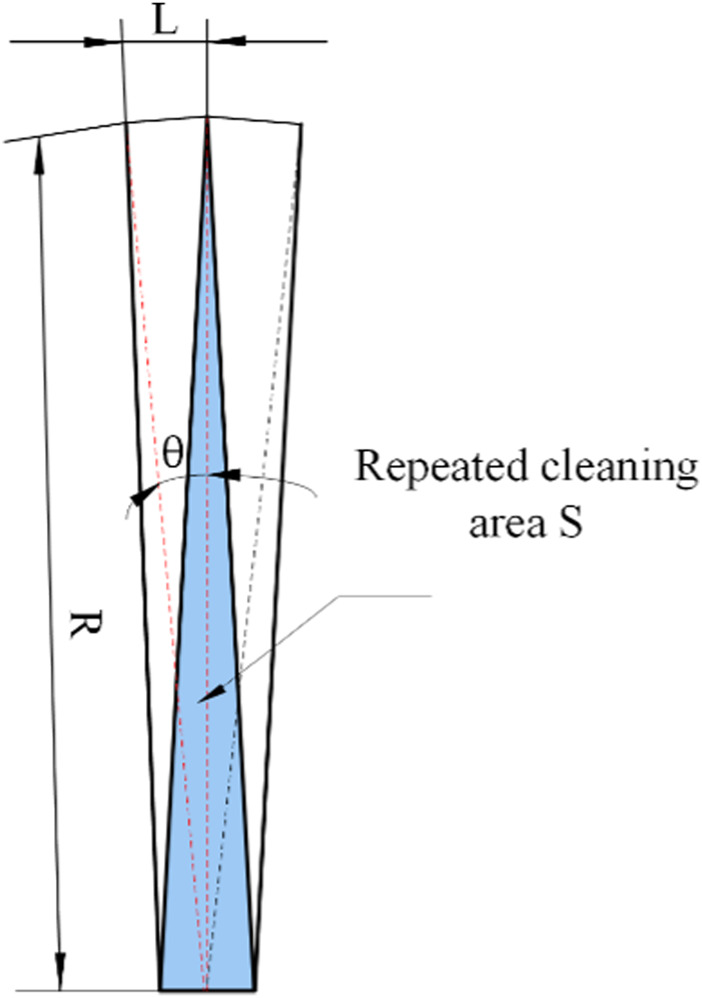
Diagram of the overlapping area of adjacent radius acquisition path.

The path can be fully covered, but in order to meet the economic cost and walking safety characteristics, path planning needs to meet the requirements of a low repetition rate. As shown in Figure 5, the overlapping area of adjacent radius paths is shown in the schematic diagram, and the middle blue is the overlapping area.

It can be seen that because the adjacent radius paths are not parallel, numbers of repeated acquisition areas will occur near the connection center area. In order to reduce the repetition rate of the acquisition path, the basic path of mining should be ensured to be parallel, so this path is not suitable for use.

#### 3.2.2 Arc round-trip acquisition path

First of all, in order to ensure that the manganese nodule mining vehicle collects and walks in the sector area, the path needs to meet the spatial constraint conditions of Eq. 5.

$$D=\left\{\left(\theta ,d_{i}\right),0^{\circ }\leq \theta \leq 60^{\circ };R_{conection\;center}\leq d_{i}\leq R_{\min ing\;area\;radius}\right\}$$ (5)

**FIGURE 6:**
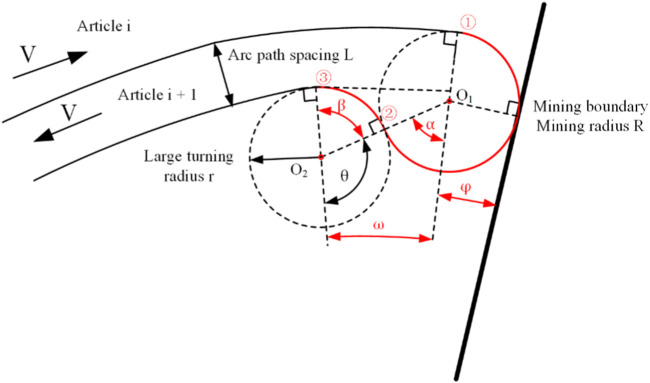
Diagram of boundary turning process of arc round trip acquisition path.

The acquisition of the manganese nodule mine car belongs to the traversal cycle acquisition. Therefore, in order to ensure the requirements of a high acquisition rate and low repetition rate, the acquisition path should meet the parallel relationship. At present, many S-shaped paths are proposed. According to the fan-shaped sub-region, the arc is used to replace the straight line as the round-trip acquisition path in this paper. Secondly, the seabed manganese nodule deposit is a thin and soft substrate, and the mining vehicle is prone to sliding and subsidence during the turning process. In order to ensure the safety of the walking operation, the speed difference between internal and external tracks in the turning process of the mining area boundary should not be too large, it is necessary to ensure that the speed ratio of the left and right tracks is less than 2:1 during the turning process of the mining vehicle. Because the propulsion speed of the mining vehicle in the seabed studio is 0.1–1 m/s, the speed difference between the left and right tracks cannot exceed 0.5 m/s. In order to ensure the acquisition rate of mining vehicles, the distance between the arc driving path after steering and the arc path before steering should not be too large, it is necessary to ensure that the turning radius of the mining truck is greater than the width of the collecting head of the mining truck, that is, r > L, where L = 2.0 m. In order to meet these two requirements at the same time, this paper combines the structural characteristics of the fan mining area to improve the S-type path, and proposes a new type of manganese nodule mining vehicle acquisition path, as shown in Figure 6.

The mining vehicle begins to traverse and collect the arc farthest from the center of the circle in the fan-shaped mining area. The width of the acquisition head of the mining vehicle is L. Then at the *i*th path, the distance between the center line of the mining vehicle and the center of the fan-shaped circle is $d_{i}=R-\left(i+\frac{1}{2}\right)L$ . As shown in Figure 6, is the schematic diagram of the arc path turning in the boundary of the mining area. To realize this path, it is necessary to adjust the speed of ①, ② and ③ times in the diagram, and determine that the speed ratio of left and right tracks is consistent at each turning, so as to ensure that the turning radius is the same, which can reduce the complexity caused by multiple speed settings. The three-speed regulations are:

1) When the mining vehicle reaches the boundary of the mining area, it first walks towards the junction center with a large turning radius, and the arc trajectory is tangent to the boundary of the fan-shaped mining area. *O*_1_ is the center of the first turning circle, and the angle from the mining area boundary to the mining area boundary and the steering angle are *φ* and *δ* respectively when the first speed is changed. According to the triangular relationship in the figure, the following formula can be obtained:

$$\left\{\begin{array}{l} \frac{d_{i}-r}{\sin\frac{\pi }{2}}=\frac{r}{\sin\varphi }\\ \cos\alpha =\frac{{\left(d_{i}-r\right)}^{2}+{\left(2r\right)}^{2}-{\left(d_{i+1}-r\right)}^{2}}{2\cdot \left(d_{i}-r\right)\cdot 2r} \end{array}\right.$$ (6)

The first turning angle is:

$$\varphi =\arcsin\left(\frac{r}{d_{i}-r}\right)$$ (7)

$$\delta =180^{\circ }+\alpha$$ (8)

2) After the first *δ*-angle steering, the mining vehicle makes a second steering in the same turning radius away from the junction center, *O*_2_ is the center of the second turning circle, and the angle from the mining area boundary and the turning walking angle are *ω+φ* and *β* when the second variable speed is changed; according to the triangular relationship in the figure:

$$\left\{\begin{array}{l} \frac{d_{i+1}-r}{\sin\alpha }=\frac{2r}{\sin\omega }\\ \beta =\alpha +\omega \end{array}\right.$$ (9)

3) After the second turning *β* angle, the mining vehicle starts the third turning and turns to the arc acquisition path; the arc path after three variable speeds is parallel to that before the variable speed, and the spacing between the two is controlled to be the width of the mining vehicle.

Taking into account the fluid disturbance characteristics of seabed soil, mining vehicles should minimize the turning characteristics, so the arc round trip acquisition path is more suitable for fan mining areas.

## 4 Modeling of mining terrain environment

The terrain of the ocean seabed manganese nodule mining area is relatively flat, but there are still some obstacles that cannot be accessed by tracked vehicles, including steep slopes, high sea rocks, and ravines that cannot be crossed. Therefore, it is necessary to extract terrain geometric parameters according to the DEM elevation map of the manganese nodule mining area and analyze the normal driving of mining vehicles, mainly including terrain slope and relief.

### 4.1 Extraction of terrain slope in a mining area

**FIGURE 7:**
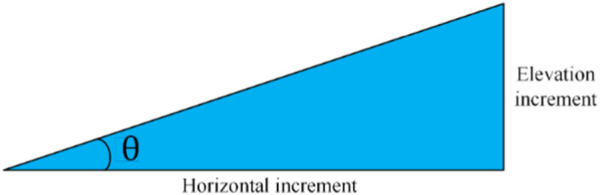
Definition of terrain slope.

**FIGURE 8:**
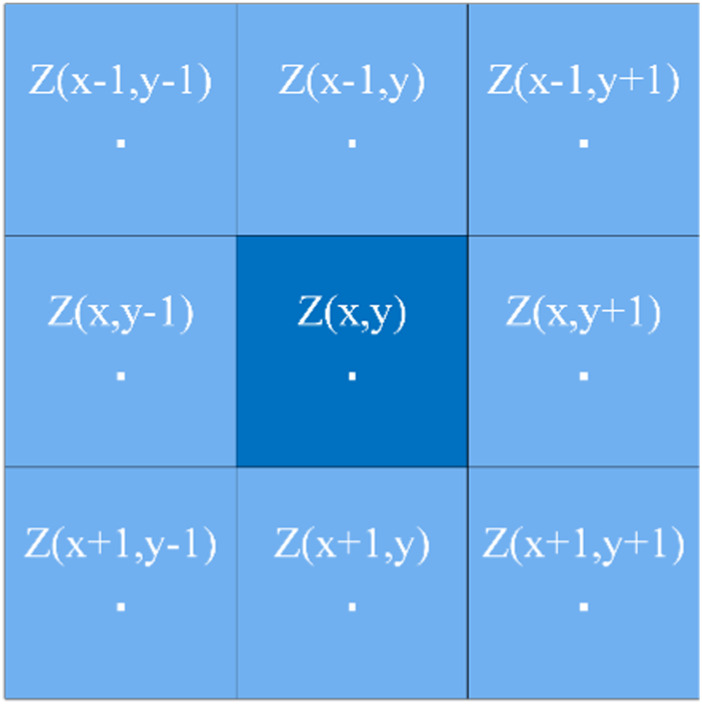
Slope convolution calculation template.

In a broad sense, the slope of a point is defined as the angle between the tangent plane and the horizontal plane of the point, indicating the inclination of the terrain here, as shown in Figure 7. Therefore, the slope is a vector with direction, and the size is determined by the elevation change rate of the central pixel in the horizontal ( $\frac{dz}{dx}$ ) and vertical ( $\frac{dz}{dy}$ ) directions. At present, there are many methods to solve the slope, among which the fitting surface method is more commonly used and the calculation results are good. As shown in Figure 8, with the required slope point as the central pixel, and combined with the surrounding eight neighborhood pixels, a 3*3 convolution is formed. The horizontal increment and elevation increment are determined by the 3*3 pixel, and then the slope value is obtained by fitting. The formula is as follows:

$$slope_{x}=\frac{1}{8x_{resolution}}\left\{\begin{array}{l} \left[f\left(x-1,y-1\right)+2f\left(x-1,y\right)+f\left(x-1,y+1\right)\right]-\\ \left[f\left(x+1,y-1\right)+2f\left(x+1,y\right)+f\left(x+1,y+1\right)\right] \end{array}\right\}$$ (10)

$$slope_{y}=\frac{1}{8y_{resolution}}\left\{\begin{array}{l} \left[f\left(x-1,y-1\right)+2f\left(x,y-1\right)+f\left(x+1,y-1\right)\right]-\\ \left[f\left(x-1,y+1\right)+2f\left(x,y+1\right)+f\left(x+1,y+1\right)\right] \end{array}\right\}$$ (11)

Where *x*_*resolution*_ and *y*_*resolution*_ are the resolutions of the unit pixel in DEM elevation data, and the vector sum of two vertical slope components is equal to the slope vector of the point, namely:

$$slope=\arctan\sqrt{slop{e_{x}}^{2}+slop{e_{y}}^{2}}$$ (12)

The slope is usually measured in degrees, so

$$slope_{\deg}=slope\cdot 57.29$$ (13)

Slope direction

$$aspect=slope_{x}/slope_{y}$$ (14)

### 4.2 Extraction of topographic relief in mining area

Terrain relief is a measure of the degree of relief of a point in the terrain, usually calculated by the variance of elevation value in the neighborhood of the point. In this paper, the eight-neighborhood point method mentioned above is still used to analyze the whole region using the moving pixel. The variance of elevation value measures the topographic relief in the neighborhood *U*_*i,j*_ with point *p* (*i,j*) as the center. The formula is as follows:

The average elevation in the neighborhood of point *p* (*i,j*) is:

$${\bar{f}}_{\Phi_{i,j}}=\frac{\sum_{\left(x_{a},y_{b}\right)\in U_{i,j}}f\left(x_{a},y_{b}\right)}{N_{U_{i,j}}}$$ (15)

The variance of elevation value is:

$$\sigma_{\Phi_{i,j}}=\frac{\sum_{\left(x_{a},y_{b}\right)\in U_{i,j}}{\left\{f\left(x_{a},y_{b}\right)-{\bar{f}}_{\Phi_{i,j}}\right\}}^{2}}{N_{U_{i,j}}-1}$$ (16)

In the formula: $\left(x_{a},y_{b}\right)\in U_{i,j}$ ; $N_{U_{i,j}}$ a Potential for a collection *U*_*i,j*_.

According to the design criteria for manganese nodule mining vehicle capacity, the threshold method is used to determine the obstacles that cannot be crossed in the mining area, and the three-dimensional topographic map of the mining area is converted to a two-dimensional traffic evaluation data model.

## 5 Path planning algorithm design

### 5.1 Constraint condition analysis of mining vehicle

Firstly, according to the shape characteristics of the mining area, the arc and radius are divided to generate all paths in the mining area.

Radius route angular resolution:

$$angle\_resolution=\left(\max\_angle-\min\_angle\right)/N_{1}$$ (17)

Distance resolution of arc route:

$$rou\_resolution=\left(\max\_radius-\min\_radius\right)/N_{2}$$ (18)

Where *max_radius* is the radius of the fan-shaped mining area and *min_radius* is the radius of the center of the round seated bottom connection; where *max_angle* is the maximum angle of the sector mining boundary selected and *min_angle* is the minimum angle of the sector mining boundary selected; All arc paths in the mining area are generated by the resolution of *angle_resolution* size, and the walking path interval of the mining vehicle is set to be *differ_L = i·angle_resolution*. Where *i* is solved according to the width of the mining head, and in order to ensure that the mining vehicle can be docked with the next path after a large turning, the walking interval of the mining vehicle and the turning radius must ensure the following relationship:

$$\left(radius\cdot 2>differ\_L\right)$$ (19)

According to the resolution of the fan-shaped mining area, the distance between the first path and the center of the mining area is:

rou=min⁡_radius+rou_resolution⋅route_idx (20)

Where *rou* is the radius length of the current arc path; where *route_idx* is count all paths from the connection center; The angle of each point in the collection path relative to the origin of the connection center is:

angle_arr=angle_arr⋅angle_resolution+min⁡_angle (21)

According to the turning constraints, the first turning angle:

$$\left\{\begin{array}{l} a=rou-radius\\ b=2\cdot radius\\ c=rou+differ\_L-radius \end{array}\right.$$ (22)

$$temp=\left(a^{2}+b^{2}-c^{2}\right)/\left(2ab\right)$$ (23)

$$angle1=\arccos\left(temp\right)$$ (24)

Second turning angle:

$$angle2=\arcsin\left(radius/\left(rou-radius\right)\right)$$ (25)

Where *radius* is a Large turning radius.

The arc length of the mining vehicle during the two-speed regulation processes is:

$$arc\_length=rou\cdot \left(angle1+angle2\right)$$ (26)

### 5.2 Principle of obstacle avoidance algorithm for mining vehicle

According to the extraction of terrain indicators, the obstacle position that cannot be crossed by the mining vehicle in the terrain of the reconstructed mining area is determined to be unable to walk regional academic. Because there are few obstacles in the occurrence area of manganese nodules, and the turning angle is small when avoiding obstacles. At the same time, in order to ensure the coverage rate of the mining area as much as possible, the mining vehicle does not use a large turning radius to avoid obstacles but follows the midline theorem. When the arc path is blocked by obstacles, the mining vehicle goes back to the previous arc path along the radius direction. If the previous arc path is still blocked by obstacles, it continues to walk upward along the radius until an arc path without blocking is optimized. At the same time, the optimization is continued according to the angular resolution until the nearest non-blocking radius path is returned to the original acquisition path. Therefore, the obstacle avoidance principle conforms to the midline theory and continues to collect along the original path after obstacle avoidance, so as to maximize the mining rate. As shown in Algorithm 1.

Algorithm 1 Obstacle Avoidance algorithm.

Observer parameters: $max_{radius}$ , $\min_{radius}$ , $differ_{L}$

Calculation of distance resolution of arc route: $rou_{radius}\leftarrow \left(max_{radius}-\min_{radius}\right)/N_{2}$

**while** cycle i in [0, $rou_{radius}$ ]
  **if**: $next_{position}$ is Mining edge
   **if** turn times are 1
   
$a\leftarrow rou_{radius}$

   
$b\leftarrow rou_{radius}+differ_{L}$

   
c←2*radius c
   
$temp\leftarrow \left(a^{2}+b^{2}+c^{2}\right)/\left(2ab\right)$

   
$angle1\leftarrow \arccos\left(temp\right)$

   **end if**
   **if** turn times greater than 1.
     
$angle2\leftarrow \arcsin\left(radius/\left(rou_{radius}\right)\right)$

   **end if**
  **end if**
  
$arc_{length}=rou\left(angle_{1}+angle_{2}\right)$

**end while**

### 5.3 Simulation results of path planning

**FIGURE 9:**
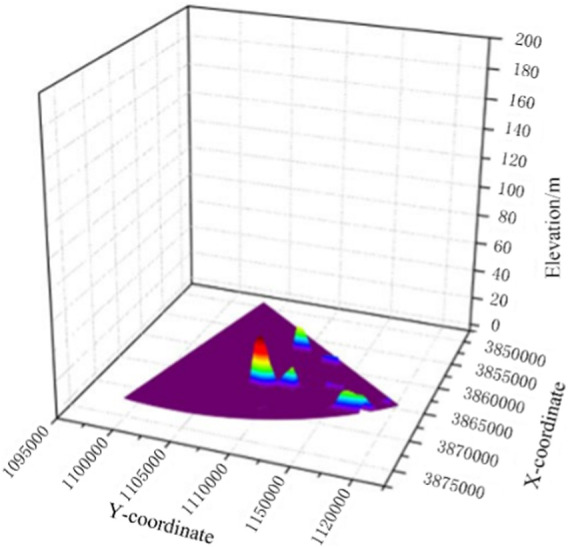
Reconstruction 3D topographic map of sector mining area.

According to the exploration contract of the polymetallic nodule mining area signed by China between the two fault zones (C-C zone) in the eastern Pacific basin and Kolarian-Kripaton, this paper extracts some DEM topographic maps from the KW-1 area of the western and eastern mining areas of the exploration contract, and uses Global map software to divide the extracted circular mining area into six parts, and selects one of the 60° sector areas as the simulation terrain data for environmental modeling. The selected circular DEM has a large range of data, a 60° sector with a radius of 25,000 m, and a resolution of 40 m × 40 m. Therefore, in order to reasonably display the topography of the mining area and facilitate the subsequent algorithm optimization, the data are first simplified and reconstructed. The threshold method is used to reconstruct the terrain data with an elevation below −5,238 m so that it conforms to the terrain characteristics of the deep-sea manganese nodule mining area. The reconstructed 3D topographic map is shown in Figure 9.

**FIGURE 10:**
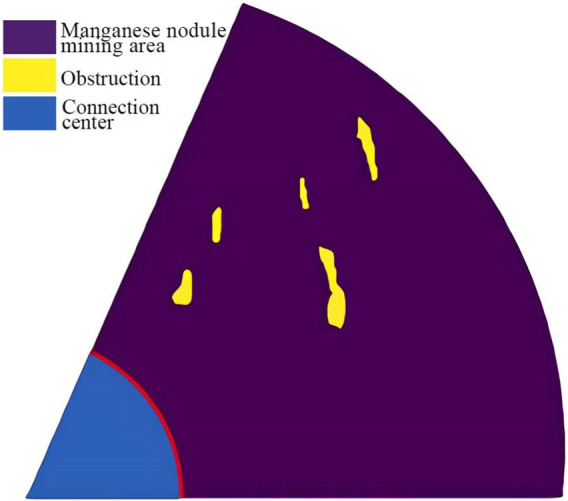
Two-dimensional data model of sector mining area..

**FIGURE 11:**
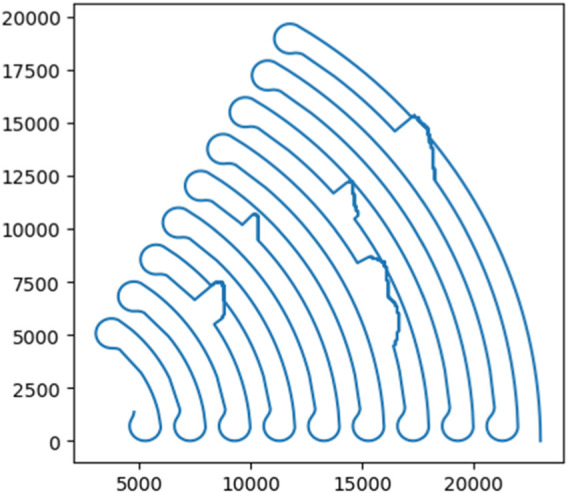
Road map of sector mining area acquisition.

The two-dimensional data model of the fan-shaped mining area is shown in Figure 10. According to the size of the fan-shaped region and the above path generation process, in the simulation experiment, the angular resolution N_1_ of the radius route is set to 10,000, and the distance resolution N_2_ of the arc route is set to 1,000. In order to adapt to the terrain data, the difference *differ_L* of two mining paths is set to 1,000, and the *radius* of the turning radius is set to 700. Combined with the principle of the obstacle avoidance algorithm in this paper, the mining vehicle starts to sweep along the arc path from the farthest distance between the fan-shaped mining area and the connection center. When it reaches the boundary of the mining area, it conducts three large turning radius 180° turning and enters the next arc path with interval L. The obtained fan-shaped mining area acquisition route map is shown in Figure 11.

From the simulation results above, it can be found that the mining vehicle can meet the constraints, strictly follow the expected arc path in the recoverable sub-region, and realize large turning radius speed regulation at the boundary of the mining area. In addition, according to the terrain feature extraction, the obstacle area can be effectively identified and the obstacle avoidance movement can be realized. The simulation experiment verifies the effectiveness of the path planning model.

## 6 Performance evaluation of acquisition path

### 6.1 Calculation of path collection area

From the analysis of the previous section, it can be seen that the mining vehicle collection area consists of three parts, namely the arc part inside the mining area and the circular part of the left and right boundaries. Assuming that the arc acquisition path passes through N times of large turning radius turning in the sector-shaped mining area, the acquisition area of the mining vehicle can be expressed as:

$$\left\{\begin{array}{l} S_{C}=\sum_{1}^{N}\left(\alpha_{i}+\beta_{i}\right)rL+\frac{\theta }{2}\left(R^{2}-{R_{i}}^{2}\right)\\ R_{i}=R-iL \end{array}\right.$$ (27)

Where *α* is the first turning speed arc corresponding to the center angle; *β* is the second turning speed arc corresponding to the center angle; *r* is the large turning radius of the mining vehicle; *L* is the acquisition width of the mining head; *R*_*i*_ is the radius length of the *i* arc path from the junction center.

### 6.2 Acquisition area of the overlapping area of adjacent paths

**FIGURE 12:**
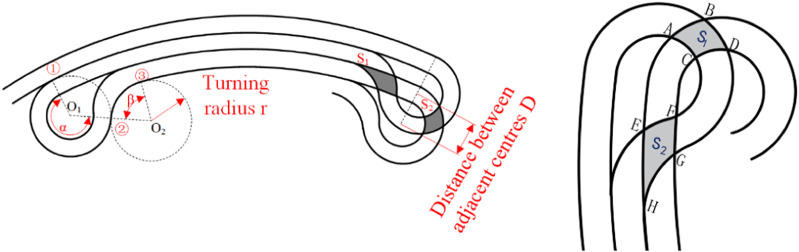
Diagram of the repeated area of adjacent paths.

Figure 12 shows the schematic diagram of two adjacent arc acquisition paths. In order to ensure that the mining vehicle can cover the whole mining area, the shadow part is the overlapping area of the two, and the two repeated areas are expressed as S_1_ and S_2_.

The rectangular coordinate system is established, and the coordinates of the intersection point of the path are obtained. Then the shadow area is integrated, and S_1_ is solved first:

Point *O*_1_ is the center of the circle, *R*_2_ is the radius of the inner circle when the mining vehicle turns, *R*_1_ is the radius of the outer circle when the mining vehicle turns, and *D* is the distance between adjacent centers.

$$\left\{\begin{array}{l} x^{2}+y^{2}={r_{2}}^{2}\\ {\left(x-D\right)}^{2}+y^{2}={r_{1}}^{2} \end{array}\right.$$ (28)

The coordinates of point *A* can be obtained from the above equation:

$$x_{A}=\frac{{r_{2}}^{2}-{r_{1}}^{2}+D^{2}}{2D}$$ (29)

Similarly, the coordinate $x_{B}=\frac{D}{2}$ of point *B* can be obtained, and then the area can be obtained by integrating the shadow part:

$$S_{1}=2\int_{x_{A}}^{x_{B}}\left(\sqrt{{r_{1}}^{2}-{\left(x-D\right)}^{2}}-\sqrt{{r_{2}}^{2}-x^{2}}\right)dx$$ (30)

Similarly, overlapping area S_2_:

$$\begin{array}{ll} S_{2} & =\int_{y_{E}}^{y_{F}}\left(\sqrt{{R_{1}}^{2}-{\left(y-L\right)}^{2}}-\sqrt{{r_{2}}^{2}-y^{2}}\right)dy+\int_{y_{F}}^{y_{G}}\left(\sqrt{{r_{1}}^{2}-y^{2}}-\sqrt{{r_{2}}^{2}-y^{2}}\right)dy\\ & \;+\int_{y_{G}}^{y_{H}}\left(\sqrt{{r_{1}}^{2}-y^{2}}-\sqrt{{R_{2}}^{2}-{\left(y-L\right)}^{2}}\right)dy \end{array}$$ (31)

### 6.3 Travel distance calculation

According to the designed arc round trip acquisition path of the mining vehicle, assuming that the arc acquisition path has undergone N large turn radius reverses in the sector mining area, the travel distance of the mining vehicle in this case can be expressed as:

$$\left\{\begin{array}{l} D=\sum_{1}^{N}\left[\frac{\pi }{3}R_{i}+r\left(\alpha +\beta +\pi \right)\right]\\ R_{i}=R-iL \end{array}\right.$$ (32)

Where *D* is the travel distance of the mining vehicle.

It is assumed that the radius of the mining area is 1,500 m, and the radius of the connection center is 200 m. The mining vehicle is mined in one of the sectors at an angle of 60°. The width of the mining mechanism of the mining vehicle is 2 m, and the turning radius is 6 m. After three times the speed regulations, the mining vehicle enters the next track from the current track. This method traverses the entire collection area. By the above calculation method, the coverage rate of collection is 99.45%, the missing collection rate is about 0.0555%, the second repeated collection rate is about 0.5129%, and the third repeated collection rate is about 0.0286%, The travel distance of the mining vehicle is 2.52 × 10^5^ m.

In order to evaluate the algorithm proposed in this paper, the algorithm proposed in this paper is compared with the traditional A* algorithm and the algorithm proposed by R Xuan, and the results are shown in Table 3 below.

**TABLE 3:** Trip distance comparison.

Algorithm	Trip distance [m]
A*	2.59 × 10^5^
The algorithm proposed by R Xuan	2.61 × 10^5^
Algorithm in this paper	2.52 × 10^5^

As shown in Table 3 above, compared with the traditional A* algorithm and the algorithm proposed by R Xuan, the proposed algorithm reduces the travel distance by 2.78% and 3.57% respectively.

The above analysis results show that the coverage rate of the path planning is large, the repeated collection rate and the missing collection rate are low, and the travel distance is shorter. The large turning radius of 6 m can ensure that the difference between the left and right tracks of the mining vehicle is small when turning. When turning, the track is basically rolled on the seabed soil rather than stirred by friction, and the repeated collection rate is low, which reduces the re-rolling of the seabed soil and ensures the structural integrity of the seabed soil. It is proved that path planning has high collection efficiency and low damage to the seabed soil environment.

## 7 Conclusion

According to the special geographical environment of manganese nodules, a new operation mode of deep-sea mining system is proposed in this paper. The whole large circular mining area is divided into six 60° fan-shaped sub-regions, and six mining vehicles continuously collect manganese nodules according to the predetermined route in the specified fan-shaped sub-region. According to the operation characteristics of the mining vehicle in each sub-fan mining area, the global traversal path planning of the mining vehicle is carried out. Considering the fluid disturbance characteristics of seabed soil, the mining vehicle should minimize the turning characteristics, so the arc round-trip acquisition path is more suitable for the fan mining area than the radius round-trip acquisition path. Through the simulation experiment, it is verified that the arc round-trip acquisition path has good obstacle avoidance ability. Through the performance evaluation, it is verified that this method has good coating coverage and a low repetition rate. Under the mining conditions set in this paper, the coating coverage rate reaches 99.45%, the missing acquisition rate is about 0.0555%, and the repetition rate is about 0.5415%. The travel distance is about 2.52 × 10^5^ m. It is an efficient path-planning method for the full coating coverage of deep-sea manganese nodule mining. At the same time, in order to better verify the feasibility of the path planning method, physical experiments will be carried out in future research.

## Data Availability

The raw data supporting the conclusion of this article will be made available by the authors, without undue reservation.
